# Automatic detection of the mental state in responses towards relaxation

**DOI:** 10.1007/s00521-022-07435-7

**Published:** 2022-06-09

**Authors:** Nagore Sagastibeltza, Asier Salazar-Ramirez, Raquel Martinez, Jose Luis Jodra, Javier Muguerza

**Affiliations:** 1 Department of Computer Architecture and Technology, University of the Basque Country (UPV-EHU), Donostia, Spain; 2 Department of Systems Engineering and Automation, University of the Basque Country (UPV-EHU), Bilbao, Spain; 3 Department of Electronic Technology, University of the Basque Country (UPV-EHU), Donostia, Spain

**Keywords:** Machine learning, Physiological computing, Mental health, Responses towards relaxation

## Abstract

Nowadays, considering society’s highly demanding lifestyles, it is important to consider the usefulness of relaxation from the perspective of both psychology and clinical practice. The response towards relaxation (RResp) is a mind-body interaction that relaxes the organism or compensates for the physiological effects caused by stress. This work aims to automatically detect the different mental states (relaxation, rest and stress) in which RResps may occur so that complete feedback about the quality of the relaxation can be given to the subject itself, the psychologist or the doctor. To this end, an experiment was conducted to induce both states of stress and relaxation in a sample of 20 university students (average age of $25.76\pm 3.7$ years old). The electrocardiographic and electrodermal activity signals collected from the participants produced a dataset with 1641 episodes or instances in which the previously mentioned mental states take place. This data was used to extract up to 50 features and train several supervised learning algorithms (rule-based, trees, probabilistic, ensemble classifiers, etc.) using and not using feature selection techniques. Besides, the authors synthesised the cardiac activity information into a single new feature and discretised it down to three levels. The experimentation revealed which features were most discriminating, reaching a classification average accuracy of up to $94.01\pm 1.73$% with the 6 most relevant features for the own-collected dataset. Finally, being restrictive, the same solution/subspace was tested with a dataset referenced in the bibliography (WESAD) and scored an average accuracy of $90.36\pm 1.62$%.

## Introduction

In recent years, the definition of health has got a new perspective focusing on the person’s physical and emotional well-being. As stated by the World Health Organization, health is not only the absence of infirmity or disease but a state in which there is a full physical, mental and social wellness [1]. Accordingly, healthcare systems increasingly demand new technologies, ranging from new medicines to complex diagnosis equipment and better preventive-health plans. Seeing these new needs, there is an increasing tendency in the area of engineering and computer science focused on developing new tools to help medical teams get either better diagnostics or reduce the costs of disease detection processes.

In this sense, several physiological computing researchers are focusing their efforts toward designing computer systems that can identify and classify patterns in the physiological signals of patients. These systems can help in traditional medicine, as for diagnosing peripheral and central nervous system diseases or disorders, and for psychological purposes such as identifying mental states by giving an automatically interpreted feedback of given physiological deviations taking place in the organism.

This study bases on the principle that living stressful or relaxing situations produces a direct reflection on the organism. More precisely, they reflect on either the sympathetic or parasympathetic parts of the autonomic nervous system (ANS). The ANS has great sensitivity, with such precision that the physiological reactions produced by responses towards relaxation (named RResp from now on) taking place in the middle of a stressful situation differ from those occurring in a calm environment.

Following these principles, many research teams have studied and published about the relationship between stress and mental health [2–4]. These days, the study of this relationship has taken importance as there has been an overall increase in the population’s stress levels as a consequence of the COVID-19 pandemic. But apart from the social perspective, this increase of stress has also triggered many disorders related to mental health [5, 6]. And, accordingly, the detection of stress onsets in humans is still a relevant topic in the scientific community [7–9].

In previous research, this team contributed to the literature by presenting an algorithm capable of detecting and classifying stress in different levels based on the information provided by two physiological signals [10]: the RR signal (which represents the time difference between two subsequent heartbeats) and the Electrodermal Activity (EDA hereinafter, which is representative of the sweating of the body). More recently, the researchers studied that an algorithm can detect the organism’s RResps by only monitoring the EDA of a person [11]. The work presented in this document continues in the same research line and aims to cover one of the future lines derived from [11]: the detection of the mental state in which the RResps take place. It is known that RResp can take place in different situations and not only during repose. Accordingly, this study has catalogued the mental contexts in which RResps can take place in three different types: relaxation happening during stress, relaxation during rest (basal) or relaxation in a sustained relaxing situation. To detect these three classes, the research team revisited the system they designed in [11] and modified it by adding new input features derived from the RR signal. The signals used for the proposed system were taken from an experiment done in the university which aimed to induce both stress and relaxation in the participants.

Concerning usability, knowing the mental state of a person when RResps occur could be useful in areas like sophrology, where professionals use the algorithm to assess how good a specific therapy and/or technology adapts to a patient and help them adjust it to obtain better results [12]. Branches of medicine other than psychiatry or psychology could also benefit detecting the mental context in which the RResps occur. For instance, certain diagnostics of neuro-physiology rely on the information of the physiological signals collected from experimental tests. Their principle is simple: the clinicians record the patient’s basal physiological values and diagnose the disease according to a comparison between the basal values and the other different physiological values recorded during the test. This explanation matches some of the experimental tests used for some neurological diseases such as Parkinson’s disease [13] or autonomic dysreflexia [14]. Subsequently, knowing the mental state of the patient when going through this type of test reveals its importance: the physiological signals collected as basal could be biased in a direction other than repose and the comparative diagnostic could lead to false results. Because of these reasons, this team presents the design of a system capable of discriminating the mental state of the person when experiencing RResps, produced either by natural or induced methods (in a higher or lower intensity).

This work is organised as follows. Section 2 describes the experimental setup of the experiment from which the researchers obtained the EDA and RR signals. Besides, it also presents the different features that were explored to see which ones are the most discriminating for the proposed objective. Then, Sect. 3 describes the process for designing the classifier for determining the mental state of the patients during their RResps. This process includes both an algorithm performance comparison and an analysis of the features extracted from the first experiment according to their capacity for discriminating between the three mental states. Besides, the best solution obtained with this process will also be tested with a reference dataset of the bibliography. The following Sect. 4 discusses the different areas in which the tool could have positive outcomes. Finally, this article presents both the conclusions reached and the future lines derived from the study in Sect. 5.

## Materials and methods

This section describes the experiment designed for collecting the physiological signals that were taken later to analysis. Apart from that, this section also covers the feature extraction process.

### Experimental setup

The researchers designed and conducted an experiment for producing stressful and relaxing situations in the participants. The sample participating in this study consisted of 20 students of the School of Engineering of Bilbao (15 male and 5 female) whose age ranged from 22 to 32 years old (average = 25.76 years, standard deviation = 3.7 years). All the students participated voluntarily in the experiment and all of them signed a consent form before taking part in the experiment where they gave consent for sharing the data collected after anonymisation. This way, the researchers collected their physiological signals and could use them to automatically detect those mental states in a later machine learning analysis.

The data collecting experiment consisted of three different stages. First, the participants had to watch a relaxing video for getting their physiology to basal levels. Then, during the second stage, the participants had to complete a 3D wooden puzzle. Third, the participants were shown again the relaxing video to see whether their physiology could calm down after the previous stressful situation. Finally, with the experiment concluded, the participants were asked to fill out a questionnaire about how they had felt during the three parts of the experiment. The first question asked them if they had felt “relaxed/normal/not relaxed at all” during the relaxing videos; the second and third questions respectively asked them about the nervousness (“nervous/normal/not nervous”) and comfort (“comfortable/normal/not-comfortable”) they had felt during the puzzle solving. After that, each participant was interviewed individually by the researchers. In these interviews, the researchers asked the participants about their feelings during the experiment. Also, they asked them about all the events that had been marked down by the researchers: puzzle piece falling, the participant drying the sweat of the hands, etc. All the information collected with the questionnaires and the interviews was used to establish the ground truth labels of the registers for the later machine learning analysis.

Regarding the collection of the signals, the researchers recorded the participants’ EDA and RR signals during the whole experiment. To this end, they used a Biopac^®^ MP36 device (sampling at 1000 Hz) and the Studentlab program (this equipment is a standard for collecting physiological signals). The signals were collected using electrodes: Two electrodes were placed in the ring and little fingers to collect the EDA [15] and a three-electrode configuration [16] was used for the electrocardiogram (from which the RR signal derives).

### The biosignals: a key for detecting the mental state

In previous research, this team proved that it was possible to detect RResps by only monitoring the changes of the EDA in [11]. As explained in the introduction, the EDA is a signal representing the capacity of the skin to conduct electricity: the conductivity of the skin will be greater when the person sweats. Sweat glands are only innervated by the sympathetic nervous system [17], which is the part of the ANS responsible for the body’s reactions to attention-demanding situations or circumstances of alert [18]. Because of it, this signal has been widely used in the area of electrophysiology for detecting a variety of mental phenomena: mental tiredness and cognitive load [19, 20], onsets of stress [21], emotional reactions [22–24], etc.

However, the researchers also noted that getting relaxed varies considerably depending on the situation in which RResps occur. In this sense, the mental state reflects on how the RResp impacts the physiology of the person relaxing Hence, this work seeks to go a step further in the analysis of RResps and detect not only an occurring RResp but also the person’s mental state at that moment. Being a signal derived from the electrocardiogram, the team added the RR signal to the study as it gives valuable information about the operation of the cardiac system (e.g., high values may show bradycardia and low values tachycardia).

In this sense, the cardiovascular system is innervated by both the sympathetic and parasympathetic branches of the ANS and thus both situations of stress and relaxation get reflected on its dynamics. Accordingly, the electrocardiogram and its derived RR signal have been widely used for the automatic detection of these affective states. For instance, relating to stress, the authors of [25] studied the different behaviour of the RR signal when a person is under mental stress, physical stress, at rest or in relaxation (they did it using Poincare’s non-linear scatter plot, which will be presented later in Sect. 2.3.3). The study of [26] gives another example, where the researchers used a combination of features coming from the electromyogram and the RR signal to detect mental stress during arithmetic calculations and Stroop tests. Then, concerning relaxation, in [27], the researchers measured the effects of meditation to reduce stress using heart-rate variability analysis (i.e., the variations of the RR signal). Besides, the researchers of [28] used both the electroencephalography and the heart-rate and RR signals to design a real-time algorithm to estimate the relaxation/meditation levels of the user. Thus, with this evidence, the authors considered that the RR signal would be of help for the classification problem addressed in this work.

**Fig. 1:**
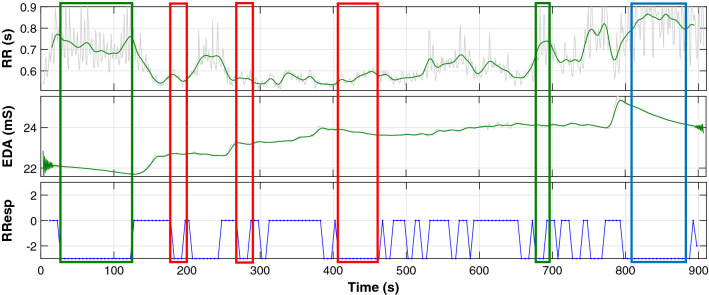
Raw (light grey) and low pass filtered RR and EDA signals (solid green) captured from a participant of the first experiment along with the RResps detected from them. RResps belonging to the different mental contexts are marked within colour boxes: blue for RResps in relaxation, green for basal RResps or in rest and red for RResps in stress

Having justified the use of the two signals for this study, an example of a register collected during the experiment is given in Fig. 1, where three different charts are shown. The first two depict the raw RR and EDA signals collected in the experiment (light grey). As it can be seen, there are eventual gaps and artefacts in the signals produced by the participant moving and making the electrodes lose contact with the skin. Hence, the team filtered the raw signals to prevent the algorithms that would be used later from mistaking decisions because of these noises (the filter will be discussed in Sect. 2.3. The filtered signals (solid green) used in this study are the ones superimposed to their raw version. On the other hand, the third chart shows the RResps that were detected in those registers using the algorithm presented in [11].

Apart from the representation of the signals themselves, Fig. 1 also depicts a good example of a person experiencing RResps in three different situations: stress, rest and relaxation. First, marked by the green box, it is possible to see that the participant was at rest (basal mental state) while watching the relaxing video of the initial stage of the experiment: the RR heartbeat time intervals were intermediate and the EDA decreased continuously without perturbation. Then, after approximately 120 seconds, the participant started the 3D puzzle-solving phase, and at this moment, the participant also began to stress. During this period, as a consequence of the sympathetic activation, both the sweating and the heart rate increased considerably. However, it is noticeable that the EDA reached a plateau around second 175 while, instead of stopping, the heart rate reached values close to the fastest of the whole experiment. These contradicting signal tendencies inside the red box indicate that the person felt eventual relaxation inside a stressful situation. Finally, around $t=800s$, Fig. 1 also depicts how the participant managed to complete the puzzle and that the experienced RResp corresponded to a relaxing situation (blue box). This time it is possible to observe that the cardiac activity of the participant reduced considerably while the EDA decreased in a very steep and continuous way. Similar situations like the three explained can be found throughout the register of Fig. 1 (some of the clearest have been marked with their corresponding box).

The analysis of Fig. 1 has shown different physiological events in which RResps could be identified. However, all of them were produced in a different mental context. Thus, the ability to detect these mental states could be useful when assessing if the relaxation taking place is genuine (relaxation in a relaxed mental state) or not (relaxation within a mental state of stress). And to do so, it is vital to extract the information contained by the physiological signals that permit discriminating better between these mental contexts.

### Feature extraction

Seeing that the signals could be used to deduce empirically the different mental states of the participants and, aiming to replicate it using an algorithm, the researchers continued their study with the feature extraction process. To this end, the researchers followed a sliding window methodology for extracting different features from the signals.

The sliding window methodology is based on chopping the signals into smaller segments as if they were pictures taken through a window using a camera. Each segment (or window) is treated separately to calculate the values of different features and after extracting all the information, the analysis is taken forward onto the next signal segment. When doing this, it is not uncommon to leave a small overlap between subsequent segments to prevent a pattern hidden in the signal from being split by two windows. This process is repeated iteratively and it gives an array or matrix with the value of each of the calculations done for every single signal segment. For the case of this study, the researchers decided that the analysis windows would have a size of 20 seconds and that they would slide it forward leaving a 5 second overlapping between consecutive segments. This decision was taken based on the properties of the two physiological signals treated in this work, which are explained in the following two paragraphs.

First, relating to EDA, researchers in the literature have used many different window sizes, going from 10s to 300s [29–32]. Besides, the authors of [33] state that features related to the phasic signal component are usually calculated using 5s time windows after the onset of the external stimulus. Moreover, they also state that features extracted from the tonic component are often computed using time windows of 20s because the upper cut-off frequency of the tonic component is around 0.05 Hz. In this sense, short-term instant EDA variations are related to the phasic component and slower variations are more related to the tonic component of the EDA. Thus, a 20s window length combines the information of both the tonic and phasic components and so it can be considered a good window size for analysing the EDA.

Nevertheless, the window size has to be appropriate for the RR too. Concerning this signal, some authors have used long time windows to treat this signal (Picard and Healey used 130s in [30]) while others have used much shorter time windows (De Santos [29] used 10s). Therefore, the team studied the signal and decided that the 20s window would be a good option for two reasons. The first reason is that the frequency spectrum of the RR signal is bounded in the [0.05–0.5] Hz range [34]. This way, the minimum frequency of the signal can be used for knowing how slow can the signal change. In this case, it would be with a period of 20 s (i.e., the chosen window size). Then, the other reason is that this size is also appropriate for analysing the EDA.

However, before chopping the signals with the sliding window approach, the team filtered first both signals using a 2 Hz cut-off low-pass filter. Using such a filter is important to remove high-frequency information and noises that were not relevant for this study. Recalling the frequencies mentioned in the previous paragraphs for the two signals, the authors considered this cut-off frequency suitable for the analysis. First, it retains the whole frequency spectrum of the RR signal. Second, it keeps untouched all the information of the EDA’s tonic component while maintaining most of the phasic component’s information.

#### Time-domain features

The first set of features extracted using sliding windows belongs to the time-domain analysis. In this process, the team extracted the 13 features listed in Table 1 for each signal window. Looking at this table, it may be noted that the last three features of Table 1 differ from the rest as they involve a ratio between different signal parameters. The reason for selecting them for the study was because they reflect how the ANS regulates the balance between the sympathetic and parasympathetic systems: when aroused, the higher frequencies of the signals vary and the surfaces contained between the instant signals and the linear regressions increase. In addition to that, it is important to note that the calculation of the features marked with “_Norm” was done after normalising the whole experiment’s RR signal according to the linear normalisation expressed in (1).

**Table 1:** Time-domain features extracted from each analysed window

Features	Description
RR_Max	Maximum value of the RR
RR_Min	Minimum value of the RR
RR_Range	Signal range of the RR: difference between maximum and minimum values
RR_Mean	Mean value of the RR
RR_Norm_Max	Maximum value of the normalised RR
RR_Norm_Min	Minimum value of the normalised RR
RR_Norm_Range	Signal range of the normalised RR: difference between maximum and minimum values
RR_Norm_Mean	Mean value of the normalised RR
RR_Slope	Slope of the linear regression of the RR in the window
EDA_Slope	Slope of the linear regression of the EDA in the window
EDA/EDA_SurfDiff	Surface between the instant EDA and its linear regression
EDA/RR_SurfDiff	Surface between the instant RR and the EDA’s linear regression
RR/RR_SurfDiff	Surface between the instant RR and its linear regression

1 $$\begin{aligned} X_{norm} = \frac{X_i-X_{min}}{X_{max}-X_{min}} \end{aligned}$$

Then, continuing with the time-domain analysis, the team also calculated the average and standard variation values of the features of Table 1. However, calculating these statistical parameters for the whole signal does not have sense because it gives only a single value for the whole register. Therefore, the team opted for iteratively calculating those statistics using only the feature values of the last four windows. By doing so, the statistical parameters based on the features extracted from each window would act as a short time memory for the recent physiological events. These 26 statistical features got the same names as the ones of Table 1 but added either “_Avg” for the average and “_Std” for the standard deviation. In total, the number of features extracted from the time-domain analysis resulted to be 39.

#### Frequency-domain features

After looking at the two signals from a time-oriented perspective, the RR signal windows were also analysed in the frequency domain. This analysis produced two more features related to the spectrum of the signal. Whereas the first feature corresponds to the frequency band that had the highest power value in all the spectrum ($P_{max}$ feature), the second feature is the frequency at which that maximum power took place ($f_{Pmax}$ feature). The power spectrum for extracting these features was calculated for the [0.05–0.5] Hz frequency range and a 0.05 Hz frequency resolution.

#### Features obtained from Poincaré’s non-linear analysis

Finally, this work also approached the RR signal from a nonlinear perspective by using Poincare’s analysis. This type of analysis is based on representing the time intervals between the electrocardiogram’s R peaks in a scatter plot. This representation of the RR times tends to produce an elliptical shape and it is possible to extract different information related to the cardiac activity by paying attention to the bisectors and other aspects related to the morphology of the ellipse [35, 36].

**Fig. 2:**
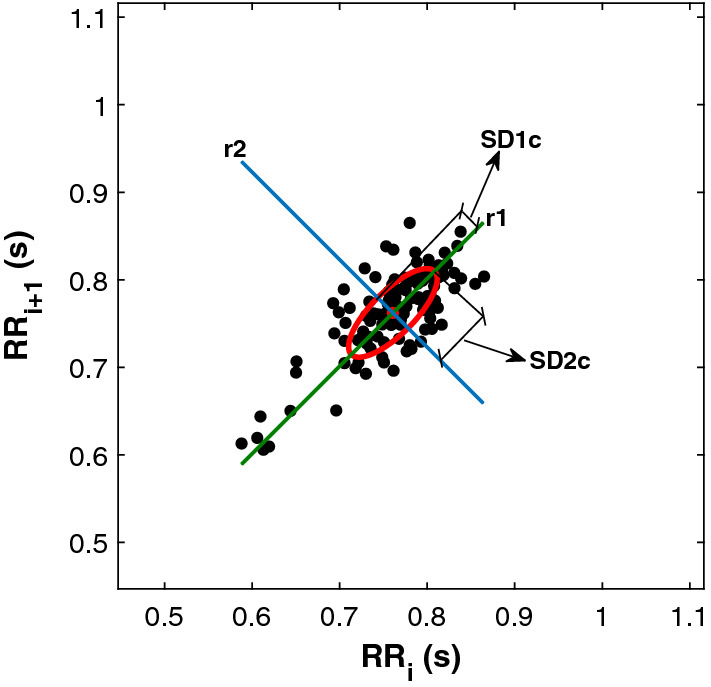
Example of the ellipse obtained from doing Poincare’s scatter plot

The first step for doing this analysis is to represent the scatter plot. To do so, assuming that the $\mathop {RR}\limits^{ \to }$ time vector has this shape $\mathop {RR}\limits^{ \to }=\left\{ RR_1, RR_2,... , RR_{n-1}, RR_n \right\}$, the points to plot would have these $\vec {x}$ (horizontal) and $\vec {y}$ (vertical) coordinates: $\vec {x}=\left\{ RR_1, RR_2,... , RR_{n-2}, RR_{n-1} \right\}$ and $\vec {y}=\left\{ RR_2, RR_3,... , RR_{n-1}, RR_{n} \right\}$. As depicted in Fig. 2, the process of representing each RR interval related to its preceding gives as result a scatter plot with an elliptical shape.

After obtaining the ellipse, the different features extracted from Poincare’s plot are derived from the mathematical analysis of the ellipse. Accordingly, it is necessary to calculate the $\left\{ x_c, y_c\right\}$ position of the centroid. This is done by calculating the average value of vectors $\vec {x}$ and $\vec {y}$: $\left\{ x_c, y_c\right\} = \left\{ {\bar{x}}, {\bar{y}} \right\}$. Knowing the centroid’s location, it is possible to calculate the distance from all the points to the centroid -expressions in (2)- and to use them to calculate the first two features, SD1c and SD2c, with the equations in (3) respectively. Concerning their meaning, SD1c represents the length of the semi-minor axis of the ellipse and SD2c the length of the semi-major axis (see Fig. 2).

2 $$\begin{aligned} d_{i}^{1}= & {} \frac{|(x_{i}-x_{c})-(y_{i}-y_{c}) |}{\sqrt{2}} \; ; \; d_{i}^{2}=\frac{|(x_{i}-x_{c})+(y_{i}-y_{c}) |}{\sqrt{2}} \end{aligned}$$

3 $$\begin{aligned} SD1c= & {} \sqrt{\frac{1}{n}\sum _{i=1}^{n}(d_{i}^{1})^{2}} \; ; \; SD2c=\sqrt{\frac{1}{n}\sum _{i=1}^{n}(d_{i}^{2})^{2}} \end{aligned}$$

Following the analysis, it is possible to split the ellipse using a straight line whose equation is $x=y$ instead of using the traditional *r*1 axis. When doing so, a new descriptor is obtained, SD1b, which was used as a feature in this study. This descriptor is obtained according to (4) and its value is almost equal to SD1c when the *n* amount of points in the plot tend to infinity.

4 $$\begin{aligned} SD1b=\sqrt{\frac{1}{2n}\sum _{i=1}^{n}(x_{i}-y_{i})^{2}} \end{aligned}$$

It is also possible to decompose SD1b into two components, SD1e and SD1d, as $(SD1b)^{2}=(SD1e)^{2}+(SD1d)^{2}$. These two components were also used as features for this study and they are obtained according to (5). It is important to note that only the $\hbox {n}_{{e}}$ $\left\{ x_i, y_i\right\}$ points located above the major bisector should be used for calculating SD1e. In the same way, the points to be used for SD1d are the $n_{d}$ points under the same bisector. Besides, the amount of $\hbox {n}_{{e}}$ and $\hbox {n}_{{d}}$ points were also used as features for Sect. 3.

5 $$\begin{aligned} SD1e=\sqrt{\frac{1}{2n}\sum _{i=1}^{n_{e}}(x_{i}-y_{i})^{2}} \; ; \; SD1d=\sqrt{\frac{1}{2n}\sum _{i=1}^{n_{d}}(x_{i}-y_{i})^{2}} \end{aligned}$$

Finally, the last two features derived from Poincare’s plot, Ce and Cd, correspond to how much did SD1e and SD1d contribute to SD1b. Being, $Ce+Cd=1$, these features can be obtained by following the expressions of (6).

6 $$\begin{aligned} Ce=\frac{(SD1e)^{2}}{(SD1b)^{2}} \; ; \; Cd=\frac{(SD1d)^{2}}{(SD1b)^{2}} \end{aligned}$$

After Poincare’s analysis, the team obtained 9 features that were used in the machine learning analysis: SD1c, SD2c, SD1b, SD1e, SD1d, $\hbox {n}_{{e}}$, $\hbox {n}_{{d}}$, Ce and Cd. And, to abridge this section, the sum of all the features extracted from the three domains produced a set of 50 features per instance of the dataset.

## Design of the classification tool

As explained in Sect. 2, the features extracted were used for automatically differentiating between the three mental states in which RResps occur. In this section, the authors present their proposal for doing such a task applying machine learning techniques. And, to be more precise, the proposal focuses on the use of classification systems for achieving this goal. This section will explain how the researchers trained different classifying algorithms for discriminating the mental state context of the participants of the experiment. Besides, this section will also present how, after analysing the initial results of the classification process, the researchers proposed a new feature that resulted to be significant for the classification: the RR_Band feature.

### Initial approach

The machine learning strategy proposed in this work poses supervised learning algorithms as candidates for classifying the mental states of the moment of RResp onsets. The database used for the machine learning analysis consisted of 1641 instances from which 586 belonged to RResps occurring when the participant was feeling relaxed (class “Relax-RResp” from now on). Then, 646 of the resting instances were fit into the “Basal-RResp” class, meaningful of the neutral mental state of repose. Finally, the remaining 409 instances corresponded to RResps taking place under mental pressure (i.e., stress) and were used for the third class: “Stress-RResp”. All these instances contained the 50 features explained in Sect. 2.3.

For getting the best possible classifier for the task, this work analysed the performance of 12 classifiers which are state-of-the-art in machine learning: 1R rule, Decision Tree (DT), k Nearest Neighbours (1-NN and 5-NN), Naïve Bayes (NB), Radial-basis Network (RBF), Support Vector Machine (SVM), Logistic Regression (LR), AdaBoost (AdaB, combining 10 decision trees), Bagging (Bag, using a combination of 10 decision trees), Random Forest (RF, 10 decision trees) and Multilayer Perceptron (MLP). This algorithm selection covers different classification paradigms, going from the simpler rule-based classification to the ensembles of classifiers. All the classifiers were built using Weka data-mining software [37] and each algorithm’s defaults settings. Besides, to improve the validity of the algorithm performance analysis, the process was conducted following the 10-run tenfold cross-validation methodology. The results of this first analysis are presented in Table 2, where the AdaB got the best accuracy ($90.51\pm 2.37$%). Besides, this algorithm also scored the best $\hbox {F}_{{1}}$ score (meaning that it has the best balance between the precision and recall performance metrics [38]).

**Table 2:** Classifier test performances using the 50 features of the puzzle’s dataset: average and standard deviations of the Accuracy (Acc.), $\hbox {F}_{{1}}$ score and Area Under the ROC Curve (AUC)

Metric	1R	DT	1-NN	5-NN	NB	RBF	SVM	LR	AdaB	Bag	RF	MLP
Acc. average (%)	73.14	86.63	88.16	83.88	71.27	74.07	77.01	76.90	$\underline{\mathbf{90.51 }}$	89.78	89.27	86.61
Acc. std. dev. (%)	3.01	2.55	2.20	2.67	3.33	3.80	3.10	3.30	2.37	2.18	2.37	2.73
$\hbox {F}_{{1}}$ average	0.73	0.87	0.88	0.84	0.71	0.74	0.77	0.77	0.91	0.90	0.89	0.87
$\hbox {F}_{{1}}$ std. dev.	0.03	0.02	0.02	0.03	0.03	0.04	0.03	0.03	0.02	0.02	0.02	0.03
AUC average	0.79	0.92	0.91	0.95	0.87	0.87	0.86	0.91	0.97	0.97	0.97	0.95
AUC std. dev.	0.02	0.02	0.02	0.01	0.02	0.02	0.02	0.02	0.01	0.01	0.01	0.01

The bold and underlined cell corresponds to the algorithm scoring the best average accuracy

However, Table 2 also shows that the Bag ($89.78\pm 2.18$%) and RF ($89.27\pm 2.37$%) got accuracies very close to that obtained by the AdaB. Moreover, the three algorithms got the same Area Under the ROC Curve metric (AUC), which gives information on how good the sensitivity and specificity of the algorithms are. Hence, it is not trivial to say which of the three is the best one and, accordingly, the team conducted a statistical analysis of the results to clarify this point. In this sense, the authors’ first step was to apply the Friedman test [39] which rejected the null hypothesis stating that all the algorithms were having equivalently accurate results (significance level of 0.05).

Then, once statistics confirmed that the algorithms were not performing equivalently, the authors conducted a second statistical test to decide which algorithm was the best. Traditionally, null hypothesis significance testing (NHST) would be used for the pairwise comparison between two competing classifiers. However, this test assumes that the observations to be compared are independent, which is not a met condition when using the cross-validation methodology. Therefore, Benavoli and his colleagues [40] proposed a more suitable alternative for these cases: the Bayesian Correlated t-test (BC test, hereinafter).

In this sense, the BC test is uses the likelihood function and the prior distribution to calculate the posterior distribution of an algorithm performing better than another. Hence, this method can be used to make pairwise comparisons between the performance metrics of two classifiers and it bases on Bayes’ rule. To calculate the posterior probability of an algorithm having better performance than the other, the method uses the generative model of the data shown in (7), where $x_{nx1}$ stands for the vector $\{x_1,x_2,x_3,...,x_n\}$ that contains the differences between the accuracies (or another metric) obtained by the two classifiers being compared along the *n* measures taken with the cross-validation process. Then, $1_{nx1}$ is just a vector of ones multiplied by the parameter of interest $\mu$. In this case, as the parameter to be compared is the accuracy, $\mu$ stands for the mean accuracy difference. Finally, $\nu \sim MVN(0, \Sigma _{nxn})$ is a multivariate normal noise, whose mean and covariance matrix $\Sigma _{nxn}$ is zero.

7 $$\begin{aligned} x_{nx1}=1_{nx1}\mu +\nu _{nx1} \end{aligned}$$

**Fig. 3:**
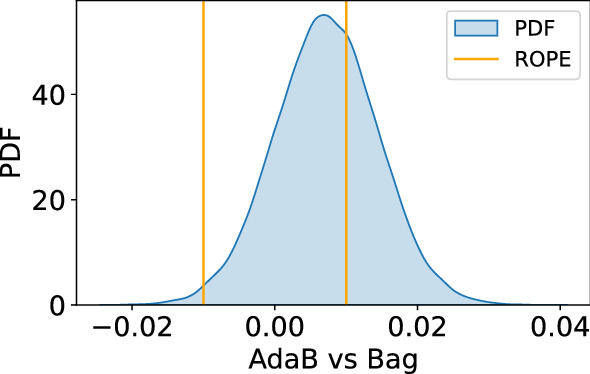
Posterior PDF for the AdaB vs. Bag comparison

Thanks to this model, Benavoli’s team [40] were able to obtain the expression for the likelihood model of the data and the posterior distribution of $\mu$, which resulted to be coincident to a Student distribution. When this is done, it is possible to compute and plot the posterior probability density function (PDF) of $\mu$ and decide whether one of the algorithms is performing better than the other. Also, the algorithms may perform equivalently from a statistical point of view. This would happen when most of the PDF fell inside the region of practical equivalence (ROPE, see [41]), which delimits the region in which the difference in accuracy between the two algorithms is less than ±1% (i.e., ± 0.01). For the sake of comprehensibility, Fig. 3 shows the posterior PDF of the BC test that compares the AdaB against the Bag (both of Table 2). In the case of Fig. 3, the 84% of the PDF is located to the right of the ROPE, which means that there is a probability of 84% that the AdaB will get better accuracy for this particular dataset.

**Fig. 4:**
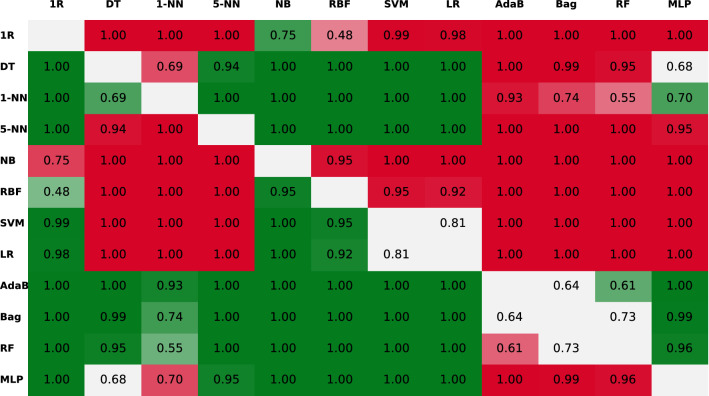
Results of the pairwise comparison using the BC test with the 50 features of the puzzle’s dataset. The values in the cells indicate the posterior probability of the comparison. The colour indicates if the algorithm in the row is better (green) or worse (red) when compared to the one in the column. The more intense the colour, the higher the probability. Grey cells indicate that the algorithms perform equivalently

Following the pattern of Fig. 3, the authors repeated this process and compared all the 12 algorithms against their 11 rivals. Nevertheless, instead of plotting all the PDFs, the results of all the BC test comparisons can be seen more clearly if they are given in the shape of a matrix (see Fig. 4), where the algorithms in the rows are compared against the ones in the columns. To be more visual, the cells in green mean that the algorithm in the row got a greater probability (the number in the cell) of getting a better accuracy than the one in the column. On the contrary, if the colour of the cell is red, then it means that the one in the row performs worse (note that the matrix is symmetric but with opposite green/red colours). Finally, the cells in light grey mean that most of the probability density is within the ROPE region and the two algorithms can be said to be performing equivalently.

Looking at the row of the Adab algorithm in Fig. 4, it is the only row that has only one cell in grey and it is therefore the algorithm winning more pairwise comparisons. Besides, in the specific case of the AdaB vs. Bag comparison, there is a probability of 64% that the two algorithms will perform similarly. Nevertheless, when the PDF is depicted (recall Fig. 3), the reader will see that almost all the remaining part of the PDF is to the right of the ROPE. Consequently, probability favours the AdaB algorithm when not performing equivalently to the Bag. Thus, the authors concluded that the AdaB was the best choice when using all the features in the dataset.

Continuing with the performance analysis, it is a common strategy to eliminate the less useful information for aiming to simplify the complexity and computational cost of the system. In this sense, not all the features have to be equally relevant when it comes to discriminating between the three mental states. Accordingly, reducing the dimension of the problem and using only the most relevant features has often a positive impact on the performance of the classifiers. Therefore, the authors tried different feature selection strategies to determine which of the 50 were the most relevant for the desired objective and the best result was obtained when using Weka’s best-first (greedy) implementation of the Correlation-based Feature Selection method (CFS) [42]. This technique chooses a feature as relevant if it correlates strongly to the class or dependent variable while correlating very weakly to the other features or independent variables. For this case of study, the CFS chose the following 11 features to be most discriminant: RR_Mean, RR_Norm_Mean, RR_Max, RR_Min, RR_Norm_Max, RR_Norm_Mean_Avg, RR_Norm_Max_Avg, RR_Norm_Min_Std, RR_Slope, EDA_Slope_Avg, EDA/EDA_SurfDiff.

The performance of the classifiers using the selected 11 features are shown in Table 3, where the RF scored the best average accuracy ($90.34\pm 2.32$%), followed by the AdaB ($90.24\pm 2.56$%) and Bag ($89.88\pm 2.33$%) algorithms. Furthermore, all three of them obtained the same $\hbox {F}_{{1}}$ value and very similar AUCs.

**Table 3:** Classifier test performances using only the 11 features selected by the CFS algorithm for the puzzle’s dataset: average and standard deviations of the Accuracy (Acc.), $\hbox {F}_{{1}}$ score and Area Under the ROC Curve (AUC)

Metric	1R	DT	1-NN	5-NN	NB	RBF	SVM	LR	AdaB	Bag	RF	MLP
Acc. average (%)	73.14	87.11	89.65	87.87	72.76	72.24	75.46	76.07	90.24	89.88	$\underline{\mathbf{90.34 }}$	81.80
Acc. std. dev. (%)	3.01	2.77	2.39	2.36	3.35	3.32	3.35	3.11	2.56	2.33	2.32	3.25
$\hbox {F}_{{1}}$ average	0.73	0.87	0.90	0.88	0.73	0.74	0.76	0.76	0.90	0.90	0.90	0.82
$\hbox {F}_{{1}}$ std. dev.	0.03	0.03	0.02	0.02	0.03	0.03	0.03	0.03	0.02	0.02	0.02	0.03
AUC average	0.79	0.92	0.92	0.97	0.88	0.88	0.85	0.90	0.97	0.98	0.98	0.93
AUC std. dev.	0.02	0.02	0.02	0.01	0.02	0.02	0.02	0.02	0.01	0.01	0.01	0.02

The bold and underlined cell corresponds to the algorithm scoring the best average accuracy

Thus, having obtained some algorithms with similar performance metric values, the authors had to conduct the same statistical tests performed before to see if the algorithms were equivalent or else which was the best. Once again, the Friedman test discarded the null hypothesis and so it could be said that not all the algorithms performed equally. Then, having rejected the null hypothesis, the authors conducted the BC test to see which algorithm had the highest probability for getting the best average accuracy. In this case, the contestants were the AdaB, Bag and RF algorithms. In this sense, the BC test stated that in their respective pairwise comparisons, the maximum probability density for all 3 algorithms would be within the ROPE limits. However, looking at Table 3, it can be seen that the RF not only scored the best accuracy but also did it with the minimum standard deviation among the 12 algorithms. For this reason, although the BC test says that the 3 had equivalent performances, the authors would opt for using the RF if they had to choose one.

Finally, seeing the results of these tests, it is also possible to conclude that there are more benefits to applying the CFS than not doing so: only extracting 11 features makes the classification easier and faster at the cost of less than 0.5% of average accuracy (AdaB(50 features) = 90.51% versus RF(11 features) = 90.34%).

### Refined approach: adding the RR_Band feature

**Fig. 5:**
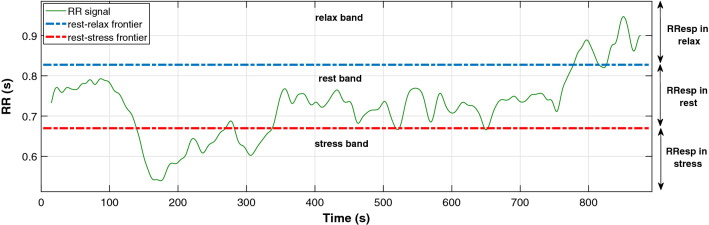
The RR_Band feature discretises the RR signal span in three bands: 1 for the relaxation band, 2 for the rest band and 3 for the stress band

After analysing the results of Sect. 3.1, the authors observed that 8 of the 11 features chosen by the CFS were related to the range and values of the RR signal: RR_Mean, RR_Norm_Mean, RR_Max, RR_Min, RR_Norm_Max, RR_Norm_Mean_Avg, RR_Norm_Max_Avg y RR_Norm_Min_Std. Therefore, the authors decided to synthesise all these features in a single feature, named RR_Band, that discretises the signal down to three values: “1-relaxation band”, “2-basal or rest band” and “3-stress band”. The boundaries for deciding RR_Band’s value in an analysis window are defined by dividing the difference between the absolute maximum and minimum of the whole signal into three equally sized bands (this is depicted in Fig. 5). Then, the value of RR_Mean is used to decide the band to which the analysed window belongs. It is important to note that, as the reference values used for calculating the boundaries depend on the person’s physiology, the RR_Band proves to be an adaptive feature robust to changes in the subject of study.

Once the value of RR_Band had been calculated for all the instances, the researchers repeated the experimentation with the same 12 classifiers to see if the new feature could improve the classification. The team decided to repeat the same process of the previous Sect. 3.1 by exploring first subspace including the 50 original features plus RR_Band and then the subspace containing only the features selected by the CFS algorithm (also including RR_Band).

The results of the first test are shown in Table 4. With a quick look at the table, the reader will see that there was a noteworthy increase in the accuracy of all the algorithms: the worst-performing algorithms of the previous two experimentations improved from scoring $71.27\pm 3.33$% (NB) and $72.24\pm 2.4$% (RBF) average accuracies to a minimum of $89.46\pm 2.11$% (NB) and $88.40\pm 2.39$% (RBF). This means that thanks to the addition of the new feature (RR_Band) the overall performance of the algorithms increased considerably, which reinforces the benefits derived from using this new feature.

**Table 4:** Classifier test performances after adding the RR_Band feature to the other 50 features of the puzzle’s dataset: average and standard deviations of the Accuracy (Acc.), $\hbox {F}_{{1}}$ score and Area Under the ROC Curve (AUC)

Metric	1R	DT	1-NN	5-NN	NB	RBF	SVM	LR	AdaB	Bag	RF	MLP
Acc. average (%)	93.36	92.52	92.97	92.35	89.46	88.40	93.36	92.22	93.90	$\underline{\mathbf{94.15 }}$	93.46	92.63
Acc. std. dev. (%)	1.86	1.65	1.57	1.91	2.11	2.39	1.86	1.76	1.67	1.64	1.76	1.93
$\hbox {F}_{{1}}$ average	0.93	0.92	0.93	0.92	0.89	0.88	0.93	0.92	0.94	0.94	0.93	0.93
$\hbox {F}_{{1}}$ std. dev.	0.02	0.02	0.02	0.02	0.02	0.02	0.02	0.02	0.02	0.02	0.02	0.02
AUC average	0.95	0.95	0.95	0.98	0.97	0.95	0.96	0.98	0.98	0.98	0.98	0.98
AUC std. dev.	0.02	0.01	0.01	0.01	0.01	0.01	0.01	0.01	0.01	0.01	0.01	0.01

The bold and underlined cell corresponds to the algorithm scoring the best average accuracy

**Fig. 6:**
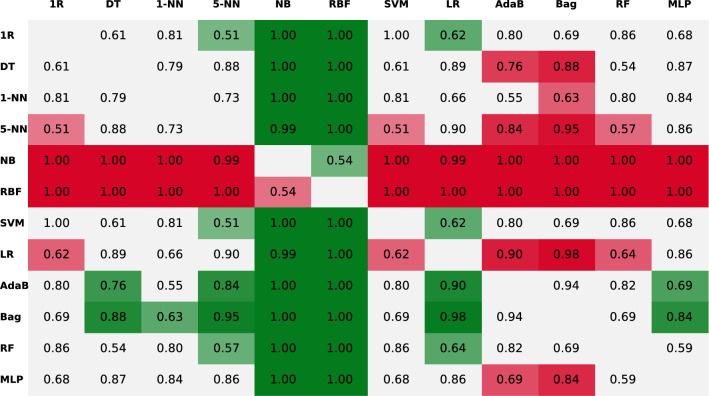
Results of the pairwise comparison using the BC test after adding the RR_Band feature to the other 50 features of the puzzle’s dataset. The values in the cells indicate the posterior probability of the comparison. The colour indicates if the algorithm in the row is better (green) or worse (red) when compared to the one in the column. The more intense the colour, the higher the probability. Grey cells indicate that the algorithms perform equivalently

In this context, the best performing algorithms were still the AdaB, Bag and RF algorithms, which scored similar accuracy, $\hbox {F}_{{1}}$ and AUC. Seeing these results, the team applied the combination of the Friedman test and the BC test to compare the performance of the algorithms. On the one hand, the former rejected the hypothesis saying the algorithms had equivalent performances. On the other hand, the latter test gave the comparison matrix presented in Fig. 6. The probabilities in this figure indicate that the Bag performed equivalently to the AdaB and the RF. Nevertheless, among the three of them, the Bag algorithm was the only one getting seven green cells, which means that it was the algorithm winning the biggest number of comparisons. Given these results, if the authors were to pick one, they would use the Bag because apart from being the algorithm that won more comparisons it was also the one scoring the best average accuracy with the minimum standard deviation in Table 4 (Bag = $94.15\pm 1.64$% vs. AdaB = $93.90\pm 1.67$% and RF = $93.46\pm 1.76$%).

Next, for the sake of consistency and as mentioned before, the authors repeated the same experiment but with the subspace including only the features selected by the CFS. As expected by the researchers, the RR_Band was among the 6 most relevant features chosen by the selection algorithm: RR_Band, RR_Mean, RR_Norm_Mean_Std, EDA/EDA_SurfDiff, EDA/RR_SurfDiff, RR/RR_SurfDiff_Std. Consequently, it can be concluded that, at least for this dataset, this feature helps to synthesise the information related to the variations of the RR signal. The results of analysing the classification problem in this subspace have been given in Table 5, where again the proposed RR_Band feature proved to help considerably in the classification. In this sense, the combination of using the new feature with the CFS algorithm obtained very similar performance metrics for all the algorithms with less than 1% of average accuracy between the best and the worst. This time, the RF algorithm resulted to be the best performing ($94.01\pm 1.73$%), getting an average accuracy very close to that of the Bag ($93.94\pm 1.75$%).

**Table 5:** Classifier test performances after adding the RR_Band feature and using only the 6 selected by the CFS algorithm for the puzzle’s dataset: average and standard deviations of the Accuracy (Acc.), $\hbox {F}_{{1}}$ score and Area Under the ROC Curve (AUC)

Metric	1R	DT	1-NN	5-NN	NB	RBF	SVM	LR	AdaB	Bag	RF	MLP
Acc. average (%)	93.36	93.38	93.18	93.46	93.29	93.06	93.36	93.26	93.71	93.94	$\underline{\mathbf{94.01 }}$	93.59
Acc. std. dev. (%)	1.86	1.97	1.92	1.85	1.87	1.81	1.86	1.82	1.86	1.75	1.73	1.75
$\hbox {F}_{{1}}$ average	0.93	0.93	0.93	0.93	0.93	0.93	0.93	0.93	0.94	0.94	0.94	0.94
$\hbox {F}_{{1}}$ std. dev.	0.02	0.02	0.02	0.02	0.02	0.02	0.02	0.02	0.02	0.02	0.02	0.02
AUC average	0.95	0.97	0.95	0.97	0.97	0.97	0.96	0.97	0.98	0.98	0.98	0.97
AUC std. dev.	0.01	0.01	0.02	0.01	0.01	0.01	0.01	0.01	0.01	0.01	0.01	0.01

The bold and underlined cell corresponds to the algorithm scoring the best average accuracy

With these results, conducting the statistical tests was crucial fairly compare the classifiers. Regarding these tests, whereas the Friedman test rejected the null hypothesis (it is highly sensitive) the BC test gave a matrix stating that all the algorithms were performing equivalently. In this sense, according to Table 5, the RF would still be the best option for the authors if they sought to get the best performance. However, these results open a new scenario in which there would not be a big impact on the performance if a simpler algorithm was used, for instance, in the implementation of a low-cost hardware device. Also, this new scenario would permit using a classifier with explanatory properties, which is an attribute often desired in medical applications (the last decision is always in the clinicians’ hands, but the algorithm’s explanation can support them taking that decision). The authors will discuss in Sect. 4 about different scenarios where using this design could be useful.

Besides, concerning the feature selection, the authors would want to recall that the RR_Band was among the most relevant features of this subspace. However, the CFS also selected the RR_Mean feature as one of the most important. This captured the authors’ attention, as they expected that the information contained by this feature should have also been covered by the RR_Band. Consequently, the researchers removed manually all the features with a correlation to RR_Band higher than 80% (including RR_Mean) to see whether RR_Band could also replace their usage and trained again all the algorithms. Nevertheless, the results of this last attempt showed that the performances decreased considerably. Therefore, the researchers concluded that although the values of RR_Mean are used for calculating the RR_Band, there is still discriminant information in RR_Mean that make it irreplaceable (it can be seen as a continuous version of the RR_Band feature).

It is also interesting to know which mental states are more complicated to classify. This information is found in Table 6, where it shows the confusion matrices for the best classifiers of the two cases studied in Tables 2 and 3 and the two of Tables 4 and 5. The results given in Table 6 show that using RR_Band improved the performance of the classifiers independently from using the CFS. Besides, these results show the robustness of the feature as it prevents the classifiers from confusing the extreme classes (i.e., “Relax-RResp” and “Stress-RResp”) with each other. Finally, it can also be seen that correctly classifying the “Basal-RResp” class is the most difficult task. It makes sense as it is the intermediate class and so it is easier for the values of the features for this class to get eventually close to the values expected for any of the other two classes.

**Table 6:**
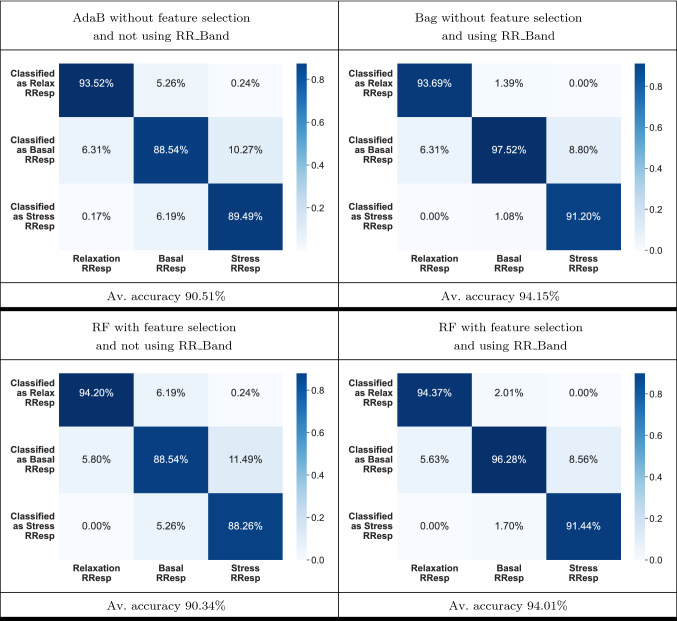
Confusion matrices of the best classifiers using the puzzle’s dataset: AdaB neither using CFS nor RR_Band, RF using CFS but not RR_Band, Bag using RR_Band but not CFS and RF using both CFS and RR_Band

### Testing the tool with a benchmark dataset

At this point, the tool presented in this paper has only been used with the own-collected dataset. Nevertheless, its wellness is to still to be verified and to do so there is no other way than to use it with a dataset other than the one used to design it. To this end, the authors looked for a dataset in the bibliography that deals with the same problem but could not find one. Instead, the authors found one that could serve this purpose: the WESAD dataset [43] from UCI’s^1^ repository. The authors chose to use this dataset for benchmarking their tool for two reasons. The first reason to do it is that it is a well-known dataset in the bibliography. Second, the physiological signals and labelling of the data contained in this dataset are similar to the dataset collected for designing the proposed tool and, accordingly, it is suitable for testing the tool presented in this paper.

The WESAD dataset contains different physiological signals from the 15 participants of the study (12 male and 3 female) with an average age of 27.5 years and a standard deviation of ± 2.4 years. In [43], the researchers used two different devices, each of them collecting certain signals. On the one hand, the first device was a RespiBan^®^ (from Plux company) and it was used to collect the electrocardiogram, EDA, electromyogram and the skin temperature from the participants’ chest. On the other hand, the researchers used an E4 wristband^®^ (from Empatica company) as the second device to collect the blood volume pulse, EDA, skin temperature and accelerometer signals of the non-dominant hand. As explained, the dataset contains several signals. However, for the comparison presented in this section, the team has only used the EDA and the RR signal (the authors of [43] derived it from the electrocardiogram and included it in the dataset).

**Fig. 7:**
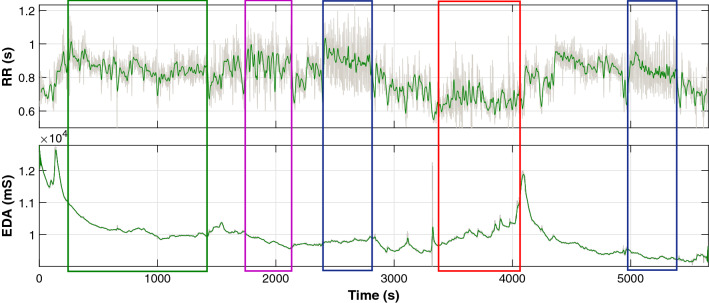
Signals of a register from the WESAD dataset. The boxes indicate the labels of the dataset: green for basal state, purple for amusement, blue for meditation and red for stress

Concerning the mental states, the experiment of WESAD consisted in taking the participants through five stages. The first stage consisted in collecting their basal signal values at rest. Then, the second and fourth stages could be either stressful or amusing; if the second was amusing then the fourth would be stressful and vice-versa. In this sense, during the amusing stages, the participants had to watch 11 funny video clips. On the contrary, during the stressful stage, the participants had to both give a 5-minute speech and then do some mathematical calculations. Finally, the third and fifth stages aimed to induce relaxation and so the participants had to practise controlled breathing meditation exercises. To give an example, a register of the dataset has been depicted in the following Fig. 7. Figure 7 shows both the EDA and RR signals along with colour boxes that represent the labels corresponding to each of the stages of the experiment (green-basal, purple-amusement, blue-meditation and red-stress).

Having presented the dataset, the team used their algorithm to detect all the RResps happening inside the labelled parts of 15 registers of the dataset. In total, the algorithm detected 4184 cases of RResp. Nevertheless, these responses could have taken place in any of the five stages of the experiment. Hence, the authors labelled each of these RResps according to the labels of WESAD. If the RResp took place in the basal stage of the experiment then the instance would get labelled as Basal-RResp. Then, the RResps detected in the stressful parts would get the Stress-RResp label and those detected during the meditation stages be labelled as Relax-RResp. It is important to note that the RResps of the amusing parts were not taken into account as they did not correspond to what was done in this article. Thus, after this process, all the 4184 had been labelled with one of the three labels used in this work: 1542 for class “Relax-RResp”, 2017 for class “Basal-RResp” and 625 for “Stress-RResp”.

Finally, the authors took the two physiological signals and processed them to extract the 6 features selected in the last configuration of the tool tuning process (recall Sect. 3.2): RR_Band, RR_Mean, RR_Norm_Mean_Std, EDA/EDA_SurfDiff, EDA/RR_SurfDiff, RR/RR_SurfDiff_Std. After doing this, they trained and tested the same 12 algorithms as before and calculated the average accuracy, $\hbox {F}_{{1}}$ score and AUC metrics (using a 10-run 10-fold cross-validation methodology). The reader can see the results of this experiment in Table 7.

**Table 7:** Classifier test performances with the WESAD dataset. The features for the classification were the 6 selected for the puzzle’s dataset

Metric	1R	DT	1-NN	5-NN	NB	RBF	SVM	LR	AdaB	Bag	RF	MLP
Acc. average (%)	55.65	86.11	84.26	82.67	63.37	66.46	65.87	68.31	$\underline{\mathbf{90.36 }}$	89.68	88.92	71.69
Acc. std. dev. (%)	2.54	1.92	1.71	1.91	1.99	2.15	1.74	1.91	1.62	1.52	1.45	2.31
$\hbox {F}_{{1}}$ average	0.55	0.86	0.84	0.83	0.61	0.64	0.63	0.67	0.90	0.90	0.89	0.71
$\hbox {F}_{{1}}$ std. dev.	0.03	0.02	0.02	0.02	0.02	0.02	0.02	0.02	0.02	0.02	0.01	0.03
AUC average	0.61	0.92	0.86	0.92	0.75	0.77	0.71	0.79	0.97	0.97	0.96	0.82
AUC std. dev.	0.02	0.02	0.01	0.01	0.02	0.02	0.01	0.02	0.01	0.01	0.01	0.03

The bold and underlined cell corresponds to the algorithm scoring the best average accuracy

The results of Table 7 show that, when using the proposed approach, the average accuracies scored by the best algorithms were close to 90% (AdaB = $90.36\pm 1.62$%, Bag = $89.68\pm 1.52$% and RF = $88.92\pm 1.45$%). These results are approximately 5% worse than the obtained with the original dataset (the puzzle’s) used for the design. Anyway, it is important to bear in mind that the data collecting experimental procedures of the two datasets were not equal. In this sense, having restricted the classification to the subspace composed of the 6 features selected for the puzzle’s dataset is one of the most exigent classification scenarios for the proposed solution. Thus, the results can still be said to be good and prove that the proposed RR_band feature and the proposed best solutions have a good generalisation capability.

**Fig. 8:**
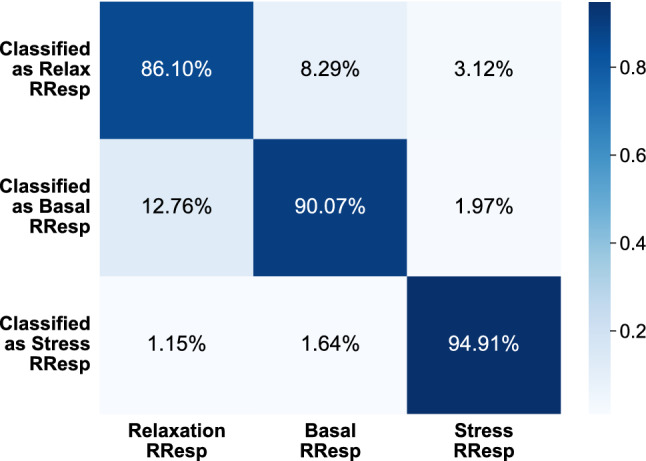
Average confusion matrix for the AdaB algorithm with the WESAD dataset. The features used for the classification were the 6 selected for the puzzle’s dataset

Finally, the authors have also calculated and presented in Fig. 8 the confusion matrix of the best performing algorithm (AdaB) when the proposed solution is applied to the WESAD dataset. From the confusion matrix it can be concluded that the instances belonging to stress were the easiest to classify and, on the contrary, the algorithm had more difficulties differentiating between the RResps occurring during relaxation (meditation) and basal state. This is the same behaviour that took place with the puzzle’s dataset.

Nevertheless, the analysis done so far has only involved the solution designed in this paper. But, to make a fair comparison, the results of this work should also be compared to those reported in WESAD’s original reference [43]. In their study, Schmidt and colleagues scored their best average accuracy using a Linear Discriminant Analysis (LDA = 93.12%) algorithm for the “stress vs. non-stress” problem. In this sense, the best results reported in [43] corresponded to a two-class problem whose classes represent opposite mental states. On the contrary, the problem addressed in this paper is a three-class problem whose classes are sub-classes of a bigger mental state (the relaxation). Accordingly, the differences between these sub-classes can be considered to be more subtle than those treated in [43]. Being so, the difference in average accuracy between the obtained in this work and the reference’s reaffirms the conclusion reached in Sect. 3.3 towards the good stability of the proposed solution and feature (RR_Band).

## Usability of the tool

As mentioned in the introduction, detecting the mental context in which people are experiencing RResps could be useful in different areas. Although its applications are majorly found in the scope of mental health, other disciplines related to medicine would also benefit from its use. In this section, the authors will discuss how the tool designed in Sect. 3 can be used in those disciplines.

To begin with the examples, the first application presented in this section belongs to the discipline of mental health and psychology. This application is the most evident one and is none other than assessing how good relaxation or sophrology techniques are performing for the specific person treated with them. For instance, the study conducted in [44] showed that using mindfulness techniques (mindfulness breathing and body scanning practice) had a positive impact on a sample of 52 college students. More precisely, they confirmed that whereas mindfulness did not affect positive emotions, it affected negative emotions by making them reduce (other studies pose the same theory, e.g., [45]). To reach these conclusions, they used different indicators such as the Five Facet Mindfulness Questionnaire (FFMQ), the Positive and Negative Affect Scale (PANAS), the State Anxiety Inventory (S-AI) or neurophysiological signal records. Specifically, the signals they collected are the heart rate (via photoplethysmography), the EDA and the pulse-rate variability.

Besides, the researchers of [44] also concluded that the benefits of short-term mindfulness training depended on the disposition towards using these techniques. In this sense, as the signals collected in this study are almost the same as in [44], the classification tool proposed in this work could be useful for assessing how good did the chosen techniques perform for each participant of the study. This application poses as a powerful tool not only for researching but also for daily use for psychologists and other professionals of sophrology. Apart from that, it would also help validate the study as it would be possible to monitor the changes in the participants’ mental state over time while putting mindfulness techniques into practice.

Then, other applications for the proposed system can be found in between mental health and traditional medicine. For instance, sophrology has been proposed for reducing the pain and anxiety levels felt by patients diagnosed with cancer [46]. In [12], Bertrand and her colleagues study the benefits of using it with patients with cancer during percutaneous interventional radiology procedures. The study used sophrology techniques with 42 patients before the radiologic intervention and the results were compared versus a control group of 18 patients who did not use them.

The results of [12] show that using relaxation techniques improved considerably both the anxiety and the pain felt by the patients: 95% of the patients using sophrology felt less anxious during the intervention compared to how they felt before it. On the contrary, although their perception of anxiety before the intervention was similar to the other group’s, 71% of the patients from the control group felt higher anxiety levels during the intervention. Furthermore, the results for the feeling of pain showed a similar tendency; the average pain perception in the control group was 4.16 points (on a scale from 0 to 10), which is significantly higher than the average 1.83 points perceived by the group using relaxation techniques. Seeing these results, it seems that using relaxation techniques benefitted the patients going through radiologic interventions. However, benefits could be maximised if the technicians performing the therapies knew which type of therapy fits best each patient. And to do so, the methodology presented in this paper could be of great help.

The last example of this section leaves mental health aside and focuses on disease diagnostics. As previously mentioned in the introduction, some diagnostics rely on comparing the values of the physiological signals collected in an experimental test. In this case, the authors give an example of how diagnosing Autonomic Dysreflexia (AD) could improve by using the mental state classifier proposed in this work. AD is an acute hypertension episode produced by the hyperactivity of the sympathetic nervous system that can have deadly consequences such as haemorrhage or brain ischemia [47]. When it comes to diagnosing AD, it is normal that the patient undergoes invasive medical tests: lumbar puncture, tilt-table testing or bladder filling processes, among others. Although it may seem too invasive, it is important to bear in mind that one of the most common causes that trigger AD onsets is the filling of the bladder [48] and so this methodology is usual in the medical study of AD. This procedure consists in comparing the basal physiological values recorded at the beginning of the experiment to the values during the rest of the experiment.

As these tests are relatively invasive, patients often feel nervous and, as a consequence, the physiological values of the initial moments of the test could be shifted towards those that would better match a stressful situation. Knowing this, it seems clear that getting to know the mental state context of the patient during the initial stages of the test could be useful for warning the clinicians about it. By doing this, the doctors could apply a corrective factor to the values registered as basal during the experiment before applying the aforementioned diagnostic comparison and, subsequently, increase the precision of the test.

## Conclusion

In this work, the authors have addressed the problem of automatically classifying three mental contexts in which a person may experience a response towards relaxation (RResp). To this end, they conducted an experiment to collect the electrodermal activity and electrocardiographic signals from 20 students to build a dataset consisting of 1641 RResp episodes of three types: Basal-RResps, Relax-RResps and Stress-RResps. From this dataset, they extracted up to 50 different features from different domains and trained a set of 12 different supervised learning algorithms for classifying the previously mentioned three classes. For this experimentation, when using the Correlation-based Feature Selection (CFS), the Random Forest (RF) performed best ($90.34\pm 2.32$%) and when not using it, the AdaBoost (AdaB) obtained the best average accuracy ($90.51\pm 2.37$%). Concerning the consistency of the results presented in this paper, all of them were validated through a 10-run 10-fold cross-validation methodology. Moreover, the best performing algorithms were selected according to state-of-the-art statistical methods (Friedman test and Bayesian Correlated t-test).

Later, the authors designed a new feature (RR_Band) that synthesises the information related to the RR signal. The team repeated the algorithm testing experiment using RR_Band and all the algorithms improved their performance when using all features (Bag = $94.15\pm 1.64$%) and when using the CFS (RF = $94.01\pm 1.73$%). Besides, when using the proposed RR_Band there was no confusion between the extreme classes. Seeing these good results, the team took the subspace of the 6 features selected for their dataset and tested it with the WESAD dataset from UCI repository. When they did it, the best average accuracy was obtained by the AdaB algorithm ($90.36\pm 1.62$%). Thus, it could be said that both the proposed solution and RR_Band feature translate robustly to datasets of other characteristics.

Finally, after analysing the confusion matrices of all the algorithms, the authors concluded that the intermediate “Basal-RResp” class was the most troublesome. Hence, to address this problem, the authors propose the future line of subdividing this class to improve the rate of errors produced in the intermediate cases. Besides, the authors would want to explore the solution in some of the areas explained in Sect. 4.

## Data Availability

The data will be made accessible upon acceptance of the article.

## Footnotes

1 Link to the dataset: https://archive.ics.uci.edu/ml/datasets/WESAD+%28Wearable+Stress+and+Affect+Detection%29.
